# The ‘chicken-egg-model’: a simple in vitro model for 3D-ultrasonography and elastography

**DOI:** 10.52054/FVVO.14.4.046

**Published:** 2023-01-27

**Authors:** M Vanthienen, K Dewilde, T Van den Bosch

**Affiliations:** Department of Obstetrics and Gynaecology, University Hospital Leuven, Belgium; Department of Development and Regeneration, KU Leuven, Belgium

**Keywords:** 3D-ultrasound, elastography, chicken-egg-model

## Abstract

**Background:**

Three-dimensional ultrasound (3D-ultrasound) and elastography are imaging techniques facilitating the diagnosis of gynaecological diseases and uterine anomalies. These non-invasive ultrasound applications may be of important added diagnostic value in gynaecological ultrasonography.

**Objectives:**

The main objective of this study was to develop a simple and cheap in vitro model for elastography and 3D-ultrasound, with an adaptable probe-target distance. The target should be visible within the model and its hardness should be modifiable. The orientation and inclination of the model should also be adjustable.

**Materials and Methods:**

We used a boiled chicken egg in a gel basis as this mimics the elastic properties of human tissues. Different boiling times were compared with the stiffness at elastography. Both 3D-ultrasonography and strain elastography were conducted with a Voluson™ E10 4-9 MHz vaginal probe.

**Results:**

Altering the eggs’ boiling times modulates the levels of stiffness, and hence the aspect on elastography.

**Conclusions:**

Our model can help trainees to practice acquisition and interpretation of elastography images. This cheap and easy-to-reproduce, in vitro “chicken-egg-model” allows for education and training of 3D-ultrasound and elastography, without causing discomfort to patients.

**What's new?:**

We created a cheap and easy to reproduce in vitro model for the education of 3D-ultrasound and elastography.

## Introduction

The use of in vitro models offers several advantages in the development and implementation of new techniques in medicine. In the development phase, in vitro models help in understanding and improving the technique. In the implementation phase, they may prove adequate training tools, allowing trainees to learn new skills without causing patient discomfort.

In gynaecology, 3D-US and elastography are emerging and promising ultrasound applications. 3D-US is gaining importance in some areas of gynaecological ultrasound, e.g., in the diagnosis of congenital uterine anomalies (Grimbizis et al., 2016). Elastography, as evaluation tool for tissue stiffness, is not yet validated in gynaecological ultrasound. Small series reported its potential benefit in the diagnosis of adenomyosis and fibroids or in the mapping of deep endometriosis (Chapron et al., 2020; Liu et al., 2018; Stoelinga et al., 2014).

We report on the development of a simple in vitro model for elastography and 3D-ultrasound. The aim was to create an in vitro model allowing optimal sound waves transmission, with an adaptable probe- target distance. The target should be visible within the model and its hardness should be modifiable. The orientation and inclination of the model should also be adjustable.

## Method

The model uses a boiled chicken egg in a gel base. For the gel base Rayher® candle wax (Rayher, Laupheim, Germany) was chosen for its elasticity properties, resembling those of human tissues. The wax contains mineral oil and polymer resin. The wax is transparent which makes it easier to locate the target object. In our model a chicken egg was chosen as target object. The object is cheap, easily available and the stiffness can be modulated according to the cooking time.

The candle wax was heated in a bain-marie until completely liquid and without any air bubbles. A square mould was first filled up the gel to 20 mm height, after which the gel solidified. In the meantime, the chicken egg was boiled in water. A cooking-time of 7 minutes was defined as “soft-boiled”, while 11 minutes cooking was defined as “hard-boiled”. After 15 minutes cooling-time the egg was peeled and put in the mould on the first gel layer. As a final step, a new layer of liquid gel was poured over the egg until completely covered. Minimum distance between the outer layer of the gel and egg was 10 mm. The covering layer can be modified to mimic different clinical situations (e.g., deep endometriosis on the vaginal wall with a distance between the probe and the lesion < 1mm or a lesion in the rectal or uterine wall > 10mm from the probe).

**Figure 1:**
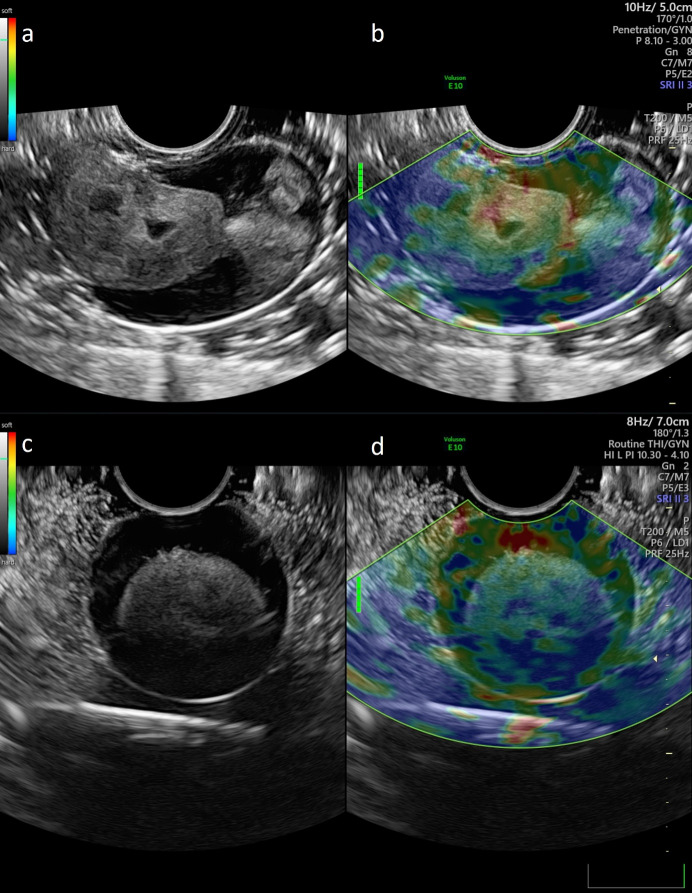
Soft boiled egg (a&b) and hard-boiled egg (c&d). On the right the elastography colour map is projected on the 2D image.

After the gel was completely hardened the next day, the ultrasound was performed using a Voluson™ E10 (GE Healthcare, Chicago, Illinois, USA) with a 4-9 MHz vaginal probe. Both 3D-ultrasonography as strain elastography was conducted on the egg model. The probe was placed perpendicular to the gel surface and a midsagittal plane of the egg was obtained. First, a 3D-volume acquisition was performed. The region of interest (ROI) and the sweeping angle were adjusted to obtain the best 3D image of the target (i.e., the boiled egg). A picture of the 3 sectional planes and the rendered 3D-volume was recorded. Subsequently, an elastogram was performed. Gentle pressure was applied at regular intervals until an optimal signal was obtained, as reflected by the quality indicator. The quality indicator provides visual feedback to compression technique and appears green when a proper technique is used (Figure 1).

## Results

**Figure 2:**
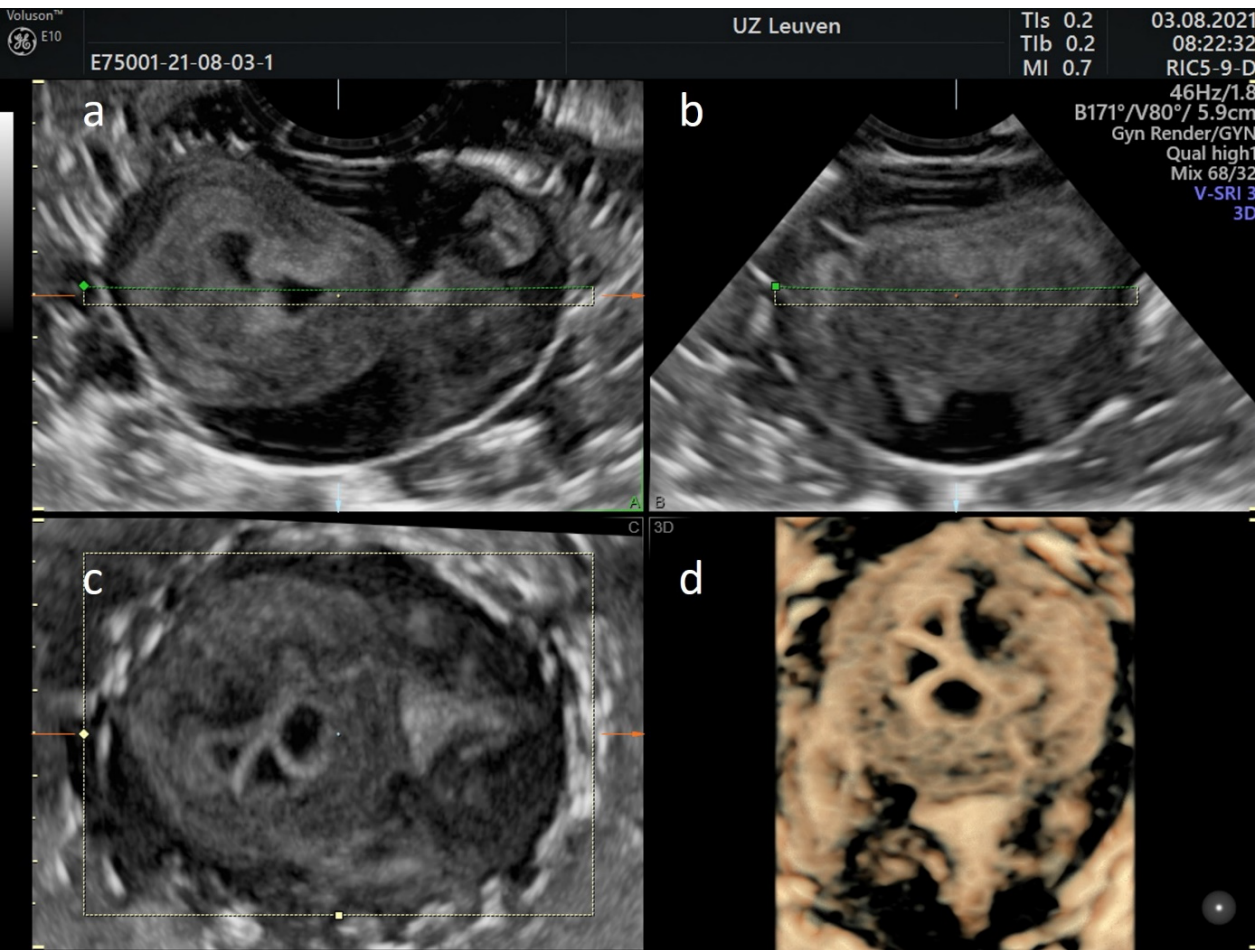
The 3D volume acquisition assigns the sagittal, transverse, and coronal planes to box a, b, and c, respectively. A rendered image is presented in box d.

Figure 2 shows a 3D-US performed on a soft-boiled egg. The 3D volume acquisition assigns the sagittal, transverse, and coronal planes to box a, b, and c, respectively. A rendered image is presented in box d. In Figure 2, the elastography was performed on both a soft-boiled (a & b) and a hard-boiled (c & d) egg. On the left the 2D-images are presented, with the corresponding elastogram superimposed on the 2D-image on the right. The green beam of the quality indicator on the left side of the elastography image (b & d) indicates optimal signal quality. The colour scale appears in the left corner of the image: it varies from red (soft) to blue (hard). Based on the chromatic scale the soft-boiled egg can be distinguished from the hard-boiled egg: in box b, the yolk has a prominent yellow/reddish tint, while in box d the yolk contains more green/blue colour.

## Discussion

We developed a simple, cheap, and effective in vitro model for 3D-ultrasound and elastography. Therefore, our model has a major didactic potential. Orientation during 3D-US is of paramount importance. For basic teaching, more geometrical and denser objects may be embedded in the gel (e.g., a paper clip, an intra-uterine device, or a bladder balloon catheter). In more advanced training, due to its texture, the egg-model offers a somewhat more complex model, more closely resembling human tissue such as an ovary or a uterus. Although offline manipulation of an acquired 3D volume is an excellent tool to learn 3D ultrasound, the interest of our model is that the trainee can acquire a volume, look at the result on screen and immediately check the orientation, comparing it to the image on the screen. The gel container’s orientation and inclination may be modulated, necessitating different probe angles to obtain optimal images.

During strain elastography external compression is applied on the tissue and the altered radiofrequency signal due to the displacement is displayed as a colour map (Ozturk et al., 2018). There is a certain lag-time between the applied external compression, the reception by the probe and the colour display. At elastography the applied pressure is very important. A standardized technique was proposed by Stoelinga et al. (2014). Realtime feedback is provided by the internal quality indicator of the ultrasound machine. Using our model, a trainee can learn to apply adequate pressure at appropriate intervals and force to obtain an optimal elastographic rendering. The fact that both the gel medium as the target organ (the boiled chicken egg) have a certain elasticity, and that the egg’s elasticity can be modulated using different boiling times makes it an even more didactic material, not only for the application of the correct external impulses, but also for a correct interpretation of the elastogram’s colour scale. Besides the boiling time affecting the egg’s stiffness, the focal depth also influences the model’s stress – strain characteristics and hence the elastography images.

Reliable non-invasive diagnosis of e.g., adenomyosis or congenital uterine anomalies is important in the workup of women with infertility, early pregnancy, paediatric and adolescent gynaecology. For this, both 3D-ultrasonography and elastography are deemed useful. Therefore, in line with the Walking Egg’s main objective, our model may prove most valuable in the development and training of diagnostic tools in a low-cost “one- stop clinic” infertility setting (Ombelet, 2013).
